# Unilateral Photopsia: An Unusual Case of Syphilitic Endophthalmitis

**DOI:** 10.7759/cureus.83984

**Published:** 2025-05-12

**Authors:** Ahmed Qaedi, Sarika Mullapudi, Tarek Persaud, Ali Hassoun

**Affiliations:** 1 Department of Internal Medicine, University of Alabama Birmingham Huntsville Regional Medical Campus (UAB Huntsville), Huntsville, USA; 2 Department of Ophthalmology, Retina Specialists of North Alabama, LLC, Huntsville, USA; 3 Department of Infectious Diseases, Huntsville Hospital, Huntsville, USA

**Keywords:** endophthalmitis, hypopyon, photopsia, syphilis, treponema pallidum

## Abstract

Syphilitic endophthalmitis represents a rare ocular manifestation that can occur in both immunocompetent and immunocompromised patients. It is often misdiagnosed due to its resemblance to many other infectious, inflammatory, malignant, and rheumatologic conditions. This report describes the case of a 66-year-old male patient who presented with visualizing bright lights in the left eye associated with a significant decline in visual acuity. A series of investigations was done, including a temporal artery biopsy with unremarkable findings. Furthermore, vitreous aspiration was performed with Gram stain revealing trace gram-positive cocci with negative culture results. Intra-vitreal vancomycin and ceftazidime were given with no symptomatic improvements. On the other hand, the serum rapid plasma reagin (RPR) titer was 1:128 with positive syphilis serologies. The patient was started on penicillin-G 4 million units intravenously every four hours for two weeks, followed by benzyl-penicillin 2.4 million units intramuscularly weekly for three weeks. Significant improvement in visual acuity was noted after completion of antibiotic therapy, with routine retinal screening demonstrating resolution of hypopyon. This case illustrates the importance of screening for syphilis in patients with unexplained changes in visual acuity, as prompt identification of syphilis and initiation of treatment are associated with favorable outcomes.

## Introduction

Syphilis is a nationally notifiable infection in the United States caused by the spirochete *Treponema pallidum* [1]. Known as the “great imitator”, its manifestations can vary widely and mimic other disease processes. It can be classified into primary, secondary or tertiary, based on several factors including time since primary inoculation, degree of infectivity, and clinical manifestations [2]. 

Over the last decade, there has been a significant rise in the incidence of syphilis in the United States, estimated as an 80% increase in reported cases from 2018 to 2022 [3]. Ocular syphilis represents a distinct clinical entity that can present at any stage, with a reported prevalence of 0.6% of total cases [4,5]. Ocular involvement can be the only presenting manifestation of syphilis, which can lead to delayed diagnosis [6]. It is estimated that approximately half of the patients with ocular syphilis are diagnosed more than one year after initial inoculation [6,7]. Delayed diagnosis and failure to initiate early treatment can lead to permanent deficits in visual acuity, including vision loss. 

A case series described six cases of ocular syphilis, of which one resulted in permanent visual deficits and another with blindness [8]. Another report found 213 cases of ocular manifestations of syphilis, with 50 cases resulting in permanent vision loss [9]. Notably, cohorts with evidence of optic atrophy prior to treatment initiation had poor visual outcomes [9]. Hence, early recognition and prompt initiation of treatment are essential to preserve visual function and prevent other end-organ damage [6]. Herein, we present a case of syphilitic endophthalmitis in an immunocompetent patient.

## Case presentation

A 66-year-old male patient, with a past medical history of type 2 diabetes mellitus, hypertension, and hyperlipidemia, presented with visualizing bright lights in the left eye associated with photosensitivity and reduced visual acuity over the past two months. The patient denied any fevers, infected skin sores, dental infections, or recent surgeries. The patient’s past ocular history consists of bilateral cataract surgical repair performed three years ago with no evidence of postoperative complications. In addition, he underwent repair of macula off retinal detachment in the right eye one year ago with an uncomplicated postoperative course. His baseline visual acuity three months postoperatively was 20/25-2 bilaterally.

Objectively, the patient remained afebrile with stable vital signs, maintaining his oxygen saturation on room air. On initial evaluation in the emergency department, visual acuity assessment using a distant Snellen chart showed 20/40 vision in the right eye. Visual acuity of the left eye was limited to hand motions only. There was no evidence of conjunctival hemorrhage or lesions. Bilateral extraocular movements were intact with no signs of nystagmus. Furthermore, there were no signs of periorbital edema, erythema, or discharge bilaterally. Other systems examinations were insignificant.

Initial laboratory investigations disclosed elevated erythrocyte sedimentation rate (Table 1). In view of the underlying suspicion of giant cell arteritis, empiric methylprednisolone was given, followed by a temporal artery biopsy. However, the temporal artery histopathology results were negative, and glucocorticoids were discontinued.

**Table 1:** Initial serum laboratory investigations at presentation WBC: white blood cell; MCV: mean corpuscular volume; Abs: absolute; BUN: blood urea nitrogen; ALP: alkaline phosphatase; ALT: alanine aminotransferase; AST: aspartate aminotransferase; CRP: C-reactive protein; ESR: erythrocyte sedimentation rate

Laboratory marker	Patient Value	Normal range
WBC	8.02 x10^3^/microliter	4.5 – 11 x10^3^/microliter
Hemoglobin	13.9 g/dL	12.2 – 16.7 g/dL
Platelets	386 x10^3^/microliter	150 – 450 x10^3^/microliter
MCV	94.6 fL	80 – 100 fL
Abs Neutrophils	5.03 x10^3^/microliter	1.5 – 7.9 x10^3^/microliter
Abs Lymphocytes	2.18 x10^3^/microliter	1.1 – 3.4 x10^3^/microliter
Abs Monocytes	0.64 x10^3^/microliter	0.3 – 1.1 x10^3^/microliter
Abs Eosinophils	0.06 x10^3^/microliter	0.0 – 0.5 x10^3^/microliter
Sodium	136 mmol/L	135 – 145 mmol/L
Potassium	4.5 mmol/L	3.5 – 5.0 mmol/L
Chloride	99 mmol/L	96 – 108 mmol/L
Carbon Dioxide	24 mmol/L	22 – 29 mmol/L
BUN	21 mg/dL	6 – 20 mg/dL
Serum Creatinine	0.7 mg/dL (Baseline 0.7)	0.5 – 1.0 mg/dL
Serum Glucose	172 mg/dL	70 – 100 mg/dL
Calcium	9.9 mg/dL	8.6 – 10 mg/dL
Anion Gap	13	7 - 17
Total protein	7.8 g/dL	6.4 – 8.3 g/dL
Albumin	4.3 g/dL	3.5 – 5.2 g/dL
ALP	99 EnzU/L	39 – 117 EnzU/L
ALT	14 EnzU/L	<42 EnzU/L
AST	15 EnzU/L	<39 EnzU/L
CRP	<0.3 mg/dL	<0.5 mg/dL
ESR	>80 mm/hr	0 – 20 mm/hr

The patient was referred to Ophthalmology for further evaluation. Slit lamp examination of the right eye showed clear anterior and posterior segments with appropriate post-surgical signs of the previous cataract and retinal detachment surgeries. No optic nerve edema or pallor was seen. In the left eye, there was evidence of hypopyon in the anterior segment with a hazy vitreous from an associated vitritis. These findings impeded visualizing the posterior segment, which precluded detailed examination, but the retina was grossly attached. Intraocular pressures were within normal limits bilaterally. A peribulbar block followed by vitreous aspiration and biopsy for Gram stain and culture was performed. The patient received empiric intravitreal vancomycin and ceftazidime along with topical moxifloxacin, gentamicin, and difluprednate ophthalmic drops. However, no symptomatic improvements were seen.

Given the high clinical suspicion of endogenous endophthalmitis, a search was done to uncover the etiologic infection. Vitreous humor Gram staining was positive for trace gram-positive cocci with negative culture results. The Gram stain finding was perceived to be a contaminant. Serum microbiological serologies were positive for *T. pallidum *(Table 2) along with elevated serum rapid plasma reagin (RPR) titers (Table 3). Blood and urine cultures did not grow any bacterial colonies. A transthoracic echocardiogram was negative for valvular vegetations. 

**Table 2:** Serum microbiological serology revealing positive syphilis serology

Serology	Patient Value	Normal Range
HIV 1 antigen and antibody	Non-reactive	Non-reactive
HIV 2 antigen and antibody	Non-reactive	Non-reactive
*Bartonella henselae* IgG	<1:128	<1:128
*Bartonella henselae* IgM	<1:20	<1:20
*Bartonella quintana* IgG	<1:128	<1:128
*Bartonella quintana* IgM	<1:20	<1:20
*Histoplasma* mycelial antibody	Negative	Negative
*Histoplasma* yeast antibody	Negative	Negative
*Histoplasma* immunodiffusion	Negative	Negative
*Toxoplasma gondii* IgM	< 3	< 9 IU/m
*Toxoplasma gondii* IgG	< 3	< 9 IU/mL
*Treponema pallidum* IgG and IgM	Reactive	Non-reactive

**Table 3:** Changes in serum rapid plasma reagin titers at initial presentation and at subsequent intervals after initiation of treatment RPR: rapid plasma reagin.

Time from diagnosis	RPR titer	Normal range
Initial presentation	1:128	Non-reactive
2 months	1:32	Non-reactive
6 months	1:16	Non-reactive
8 months	1:8	Non-reactive
12 months	1:4	Non-reactive
16 months	1:4	Non-reactive

**Figure 1:**
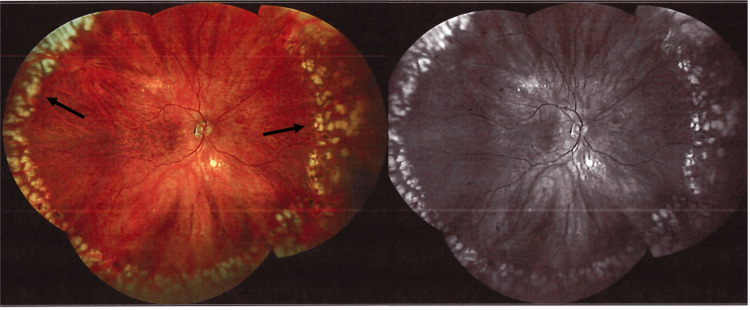
Widefield fundus and autofluorescence images of the right eye two years after treatment, demonstrating peripheral laser scars (arrows) from prior retinal detachment surgery and normal myopic changes with no evidence of optic nerve pallor.

**Figure 2:**
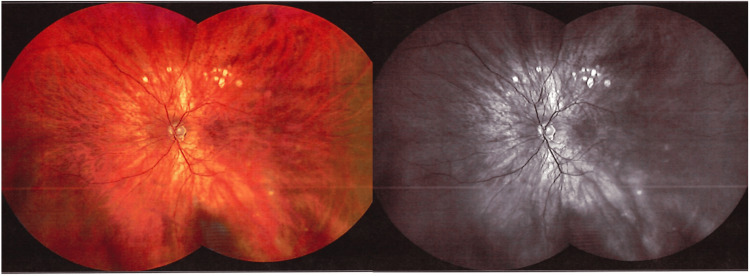
Widefield fundus and autofluorescence images of the left eye two years after treatment, demonstrating normal myopic changes with no evidence of active disease.

Treatment was initiated with penicillin-G 4 million units intravenously every four hours for two weeks, followed by benzyl-penicillin 2.4 million units intramuscularly weekly for three weeks. The patient reported significant symptomatic improvement in his vision. Subsequent routine retinal examination revealed resolution of hypopyon and associated vitritis (Figure 1, Figure 2). Repeated serum RPR titer improved to 1:4. By six months, the patient demonstrated visual acuity of 20/25+2 in the right eye and 20/25-1 in the left eye. By one year, visual acuity improved to 20/20-1 bilaterally. The patient was followed up over a period of two years with no evidence of clinical relapse.

## Discussion

Endophthalmitis represents inflammation of the intraocular vitreous and aqueous humors, mostly caused by bacterial infections. Syphilitic endophthalmitis is a rare manifestation that is under-recognized due to its resemblance to other inflammatory ocular conditions. Common risk factors include men who have sex with men and HIV positive co-infection [5], which was not present in the current case. The most common manifestation of ocular syphilis is uveitis, notably posterior uveitis or panuveitis [10,11]. The non-specific symptoms often mimic other infectious ocular pathologies such as toxoplasmosis, ocular tuberculosis, and herpetic retinitis, leading to a delayed diagnosis [10,12].

Imaging modalities such as optical coherence tomography (OCT) can be used to identify retinal abnormalities of ocular syphilis that are unable to be detected on fundus examination [10,11]. Gass et al. originally described the finding of acute syphilitic posterior placoid chorioretinitis (ASPPC), which is a characteristic but uncommon finding in ocular syphilis [13]. Delayed diagnosis is common due to the lack of specificity and low index of suspicion for syphilis. Therefore, testing for syphilis in all cases of uveitis is recommended to avoid initial misdiagnosis and ensure the best outcomes [10]. When suspecting ocular syphilis, patients should undergo both nontreponemal and treponemal testing to minimize delayed diagnosis. In addition, venereal disease research laboratory (VDRL) and fluorescent treponemal antibody absorption (FTA-ABS) testing of cerebrospinal fluid (CSF) can be considered [4]. 

In the present case, the patient did not undergo CSF analysis due to the absence of other cranial nerve dysfunction or focal neurological deficits. Additionally, the treatment regimen of ocular syphilis is similar to neurosyphilis regardless of the presence of syphilitic CSF abnormalities. We initiated treatment with intravenous penicillin-G 4 million units every four hours for a period of 14 days in accordance with the recommendations described by the Centers for Disease Control and Prevention (CDC) [14]. Unlike the treatment utilized in the case described by Lopez et al., we decided to continue antibiotics with weekly intramuscular injections of benzyl-penicillin 2.4 million units for a period of three weeks [15]. Although rare, the Jarisch-Herxheimer reaction has been previously described in the case of ocular syphilis [16]; however, it was not noted in our case.

In the presented case, there are some limitations to underline regarding the diagnosis of syphilis. Although an elevated RPR and positive syphilis serologies were seen, the patient did not demonstrate other sources of syphilitic localizations (e.g., genital). Furthermore, the source of the patient’s infection could not be determined, as he did not report engaging in sexual activity. In addition, fundus and autofluorescence images during initial presentation were not obtained secondary to the significant intraocular debris that impeded comprehensive anatomical examination. Despite these facts, the patient did demonstrate symptomatic and clinical improvements after parenteral penicillin therapy.

The prognosis of ocular syphilis is favorable with early treatment [17,18]. Our patient demonstrated significant improvements with return to baseline visual acuity by one year, which is a good outcome from an unusual disease process.

## Conclusions

Syphilitic endophthalmitis represents a rare manifestation that can present at any stage of syphilis. As a treatable cause of ocular disease, it can be missed during initial assessments secondary to its resemblance to other disease processes. The increased incidence of syphilis raises an essential component to screen for *T. pallidum* infections in cohorts with atypical changes in visual acuity and who do not respond to conventional treatments. Early diagnosis leads to improved outcomes. We presented an unusual case of syphilitic endophthalmitis that exhibited diagnostic delay but was successfully treated with no evidence of clinical relapse. Hence, healthcare professionals should be vigilant about ruling out syphilis in both immunocompetent and immunocompromised patients, as it can lead to significant morbidity, including irreversible loss of visual acuity. 
